# Supplementary figures and images for: Trackoscope: A low-cost, open, autonomous tracking microscope for long-term observations of microscale organisms
Source: PLoS One. 2024 Jul 11;19(7):e0306700. doi: 10.1371/journal.pone.0306700 (PMC11239018; doi:10.1371/journal.pone.0306700)

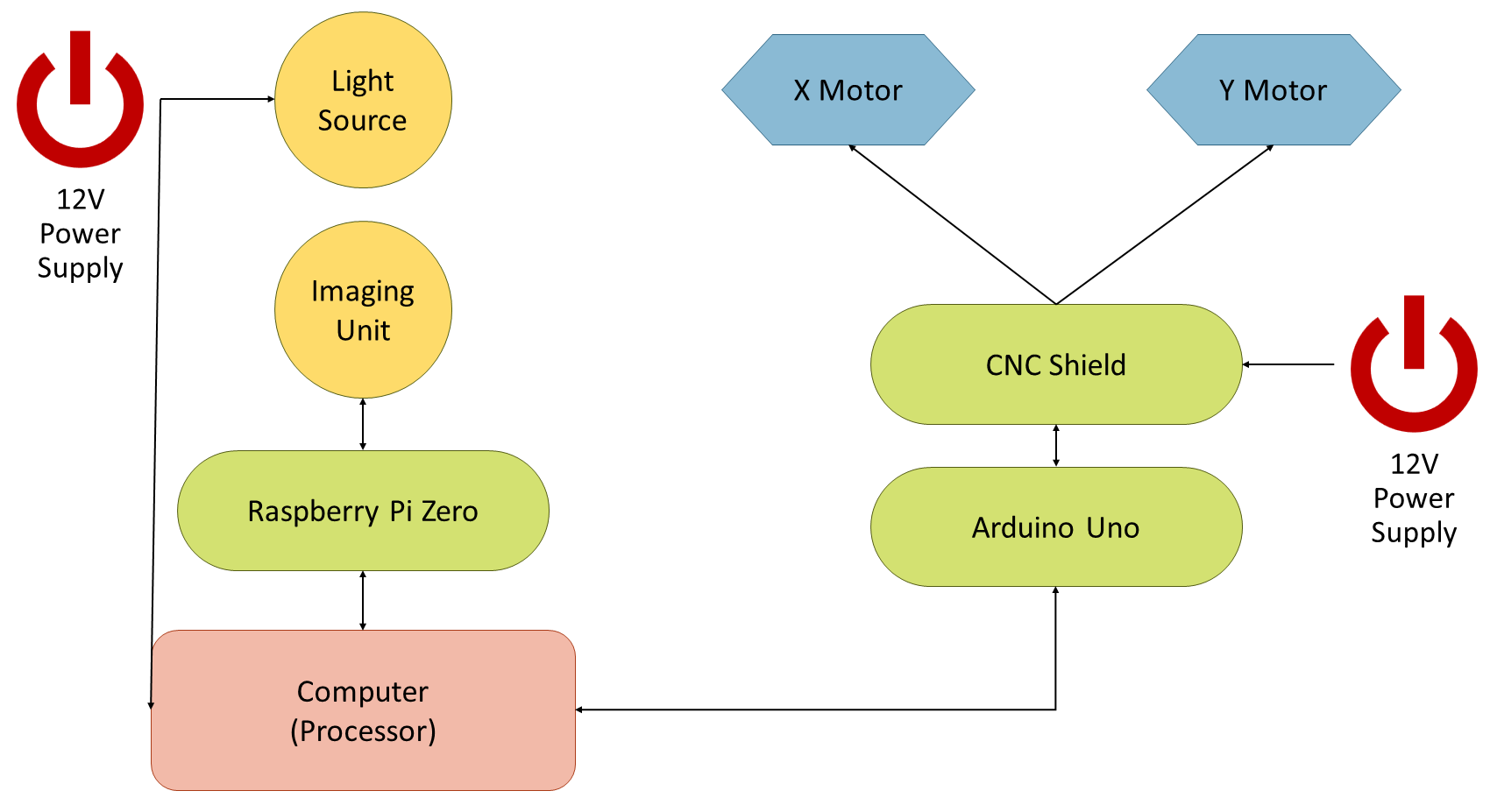

Supplement: S1 Fig — Wiring diagram for Trackoscope detailing the connections between various components. (TIF) [file pone.0306700.s001.tif]

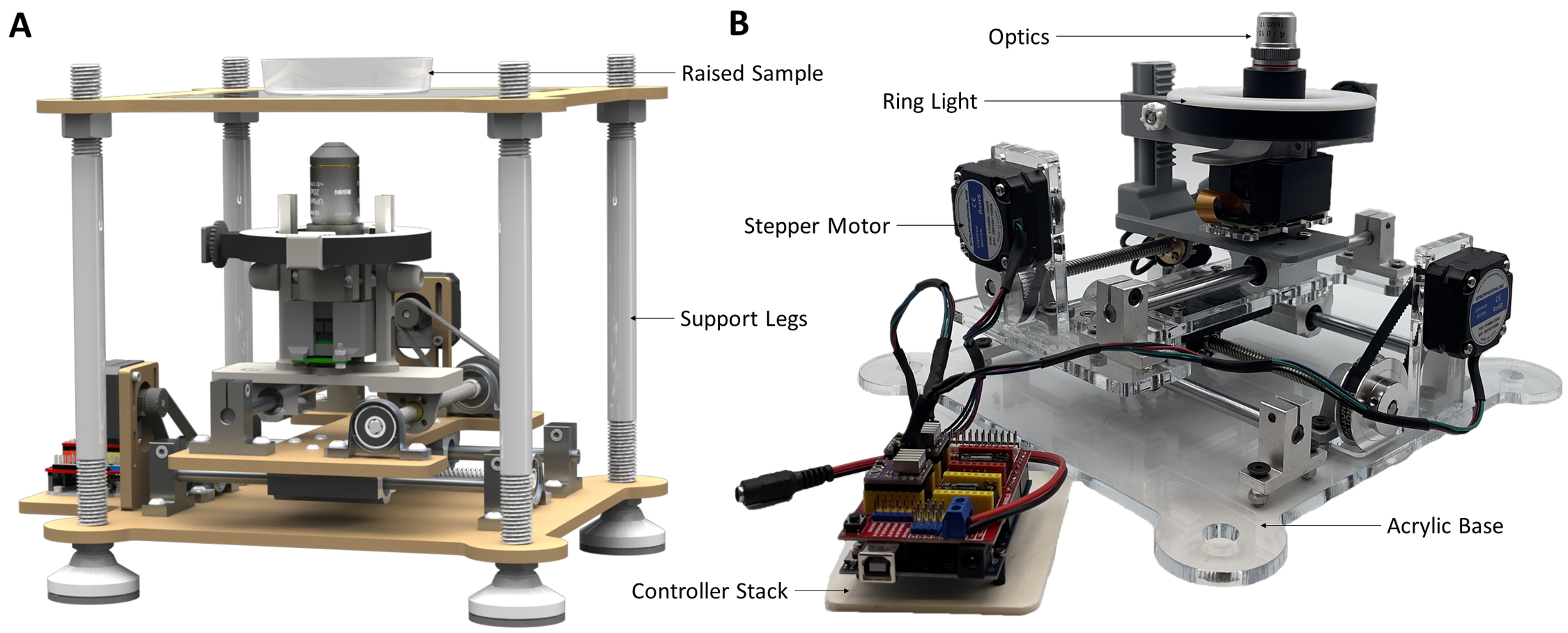

Supplement: S2 Fig — (A) CAD model of Trackoscope in the raised sample imaging setup. A white or contrasting covering is typically placed over the sample to create a clean background in the image. (B) Trackoscope is designed to be mass-produced and is constructed primarily out of laser-cut parts (acrylic or MDF) and minimal 3D printed components. It also uses standard metric nuts and bolts to join components together. (TIF) [file pone.0306700.s002.tif]

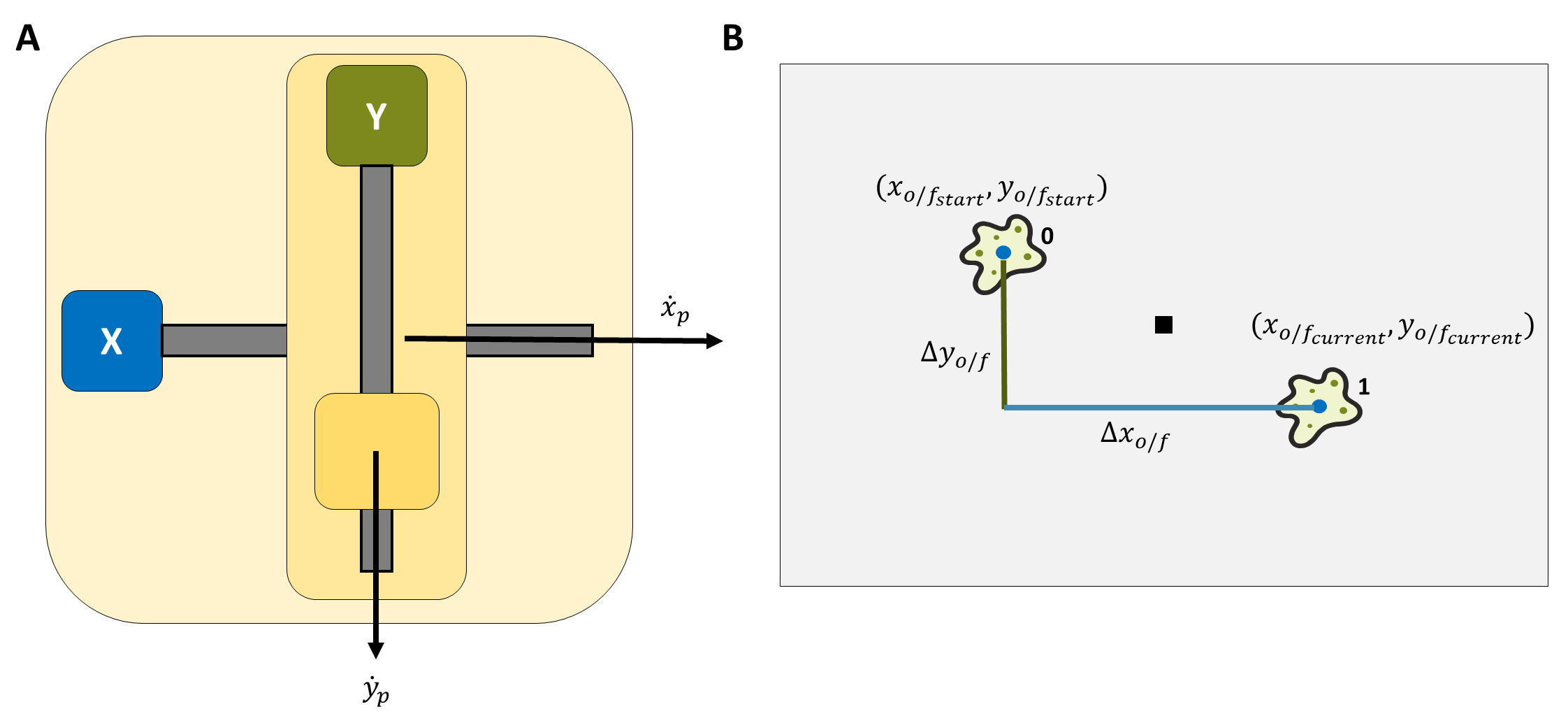

Supplement: S3 Fig — (A) (Δxo/f, Δyo/f) are calculated by taking the organism’s location in the video frame at the start (is not always (0, 0)) and finding the displacement within the frame, Δxo/f(μm)=(Δxo/fprevious+(xo/fcurrent-xo/fstart))*Cpixelstoμm, with Cpixels to μm depending on the magnification. (B) (Δxp, Δyp) are calculated by adding up all platform displacements throughout the track; for instance, a single data point would be calculated with Δxp(μm)=Δxprevious+(x˙p*50ms) where x˙ is the velocity of the axis on the platform. (TIF) [file pone.0306700.s003.tif]

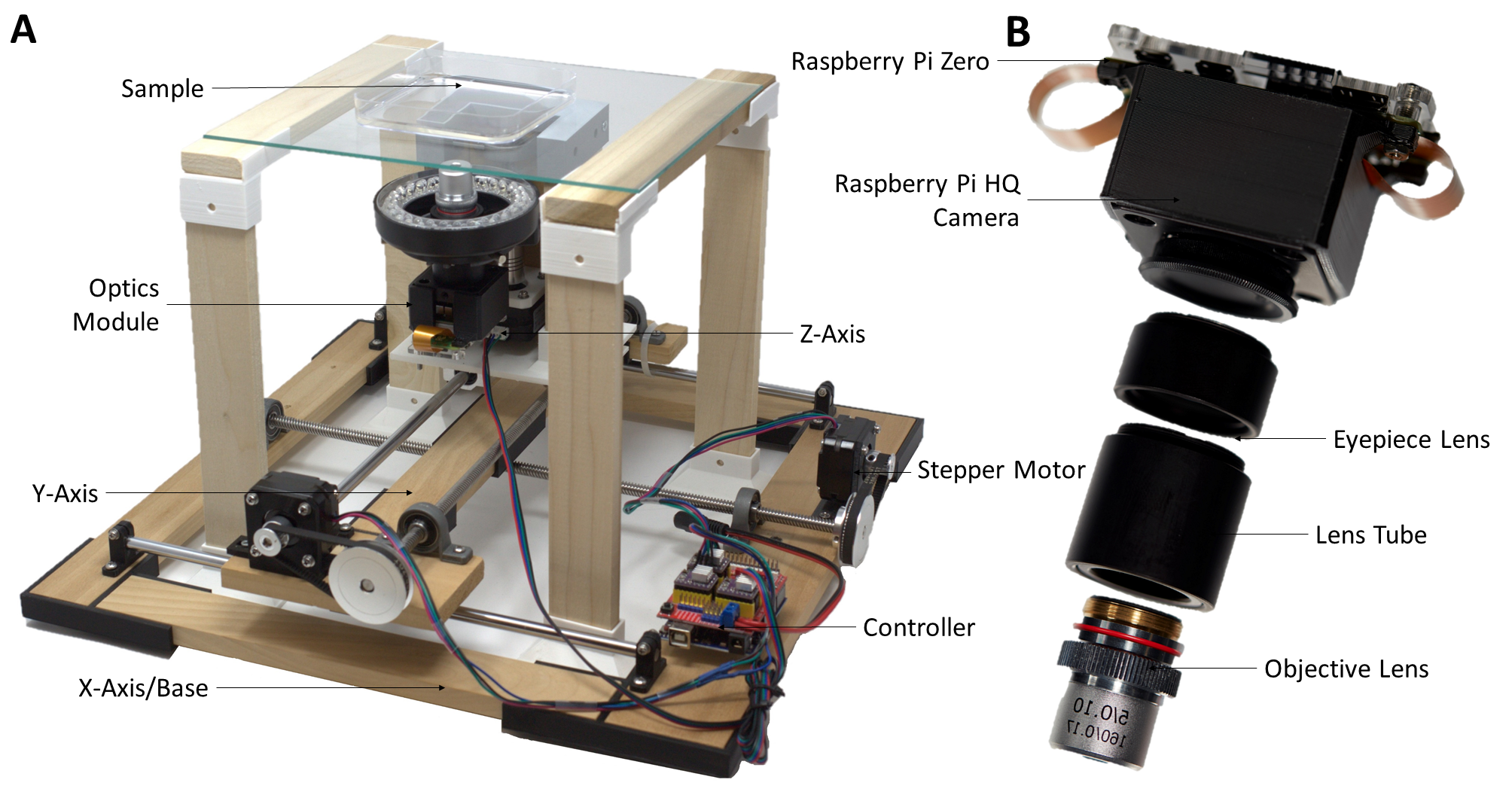

Supplement: S4 Fig — (A) The single-build customized version of Trackoscope featuring a motorized Z-axis and a raised sample that is observed from below. This prototype has a tracking area of 625cm2 (size of an A4 sheet) and is built using limited tools (hand saw, 3D-printer, and screw-drivers). (B) The physical custom digital microscope system used in both prototypes. (TIF) [file pone.0306700.s004.tif]
