# Supplementary material for: Trackoscope: A low-cost, open, autonomous tracking microscope for long-term observations of microscale organisms
Source: PLoS One. 2024 Jul 11;19(7):e0306700. doi: 10.1371/journal.pone.0306700 (PMC11239018; doi:10.1371/journal.pone.0306700)
Supplement: S1 File — More information can be found on GitRepo (https://github.com/bhamla-lab/Trackoscope). (PDF) [file pone.0306700.s012.pdf]

Supporting Information: *Trackoscope*: A low-cost, open, autonomous tracking microscope for long-term observations of microscale organisms

Priya Soneji[1], Elio J. Challita[1], Saad Bhamla[2]

1 George W. Woodruff School of Mechanical Engineering, Georgia Institute of Technology, 801 Ferst Drive NW, Atlanta, GA, 30318, USA
2 School of Chemical and Biomolecular Engineering, Georgia Institute of Technology, 311 Ferst Drive NW, Atlanta, GA, 30332, USA

Contents

***Trackoscope* part list** **2**

***Trackoscope* assembly instructions** **3**

Trackoscope part list

Part	Cost	
Arduino Uno + CNC Shield (i)	$28.88	
NEMA 17 Stepper Motors (ii)	$25.08	
Threaded Rods Set (iii)	$56.58	
Belt + Pulley (iv)	$21.98	
Legs (v)	$9.99	
Leg Screws (vi)	$3.20	**Total Cost:**
Coupling Nuts (vii)	$6.35	**$423.54**
3D Printed Parts (viii)	$4	
MDF (ix)	$5	*Actuator Cost:*
Objective Lens (x)	$14.99	*$167.05*
Achromatic Lens (xi)	$110.36	
Ring Light (xii)	$11.99	*Optics Cost:*
Raspberry Pi High Quality Camera (xiii)	$50	*$256.49*
Lens Tube (xiv)	$29.03	
Raspberry Pi Zero (xv)	$15	
Cage Plate (xvi)	$21.11	
Fasteners (xvii)	$10	

Table S1: The table lists the cost of *Trackoscope*. (i) a Shield Expansion Board V3.0 + R3 Board + A4988 Stepper Motor Driver + Heatsink, from kumantech.com, P/N K75; (ii) Nema 17 Bipolar 0.9deg 11Ncm (15.6oz.in) 1.2A 3.6V 42x42x21mm 4 Wires, from omc-stepperonline.com, P/N 17HM08-1204S; (iii) Mergorun 200mm Horizontal Optical Axis and 8mm Lead Screw Dual Rail Shaft Support Pillow Block Bearings and Flexible Shaft Coupling, from amazon.com, ASIN B06XPCY1LS; (iv) Houkr PGT2 Aluminum Timing Belt Idler Pulley Bearing 20and60 Teeth Width 8mm Born Synchronous Wheel, with a Perimeter 200mm Width 6mm Belt and a M4 Allen Wrench, from amazon.com, ASIN B081PXKKS4; (v) Antrader Pcs M10 Thread Adjustable Foot Cups Reinforced Nylon Base 48mm Diameter Articulated Feet Furniture Leg 80mm Leveling Foot, from amazon.com, P/N AZ18082701; (vi) 100mm Medium-Strength Metric Class 8.8 Steel Hex Head Screws, from mcmaster.com, P/N : 91280A198; (vii) Hitiland 5pcs Long Rod Nut Carbon Steel Hex Coupling Nuts Hexagonal Sleeve Nut Standoff M10 Threaded Fasteners, from amazon.com, P/N B07QG2YSXT; (viii) 200 grams of PLA filament; (ix) 1/4 in. x 2 ft. x 4 ft. Medium Density Fiberboard, from homedepot.com, P/N 1508104; (x) 4X Achromatic Microscope Objective, from amscope.com, P/N A4X-YX-V460; (xi) AC254-100-C - f = 100.0 mm, Ø1" Achromatic Doublet, ARC: 1050 - 1700 nm, from thorlabs.com, P/N AC254-100-C; (xii) AIXPI 4" Ring Light for Laptop with Stand and Clip, from amazon.com, ASIN B08XQCYPJ3; (xiii) Raspberry Pi High Quality HQ Camera - 12MP, from adafruit.com, 4561; (xiv) SM1 Lens Tube, 0.50" Thread Depth and SM1 Lens Tube, 1.00" Thread Depth, from thorlabs.com, P/N SM1L05 and SM1L10; (xv) Raspberry Pi Zero 2 W, from adafruit.com, P/N 5291, (xvi) 30 mm Cage Plate with Ø1" Double Bore, from thorlabs.com, P/N CP35, (xvii) M3/M4/M6 screws and nuts, from mcmaster.com, P/N 90258A187/90258A221/90258A253/90592A085/90592A090/90592A095 *All links and prices accessed January, 2023.

Assembly instructions

Trackoscope assembly instructions

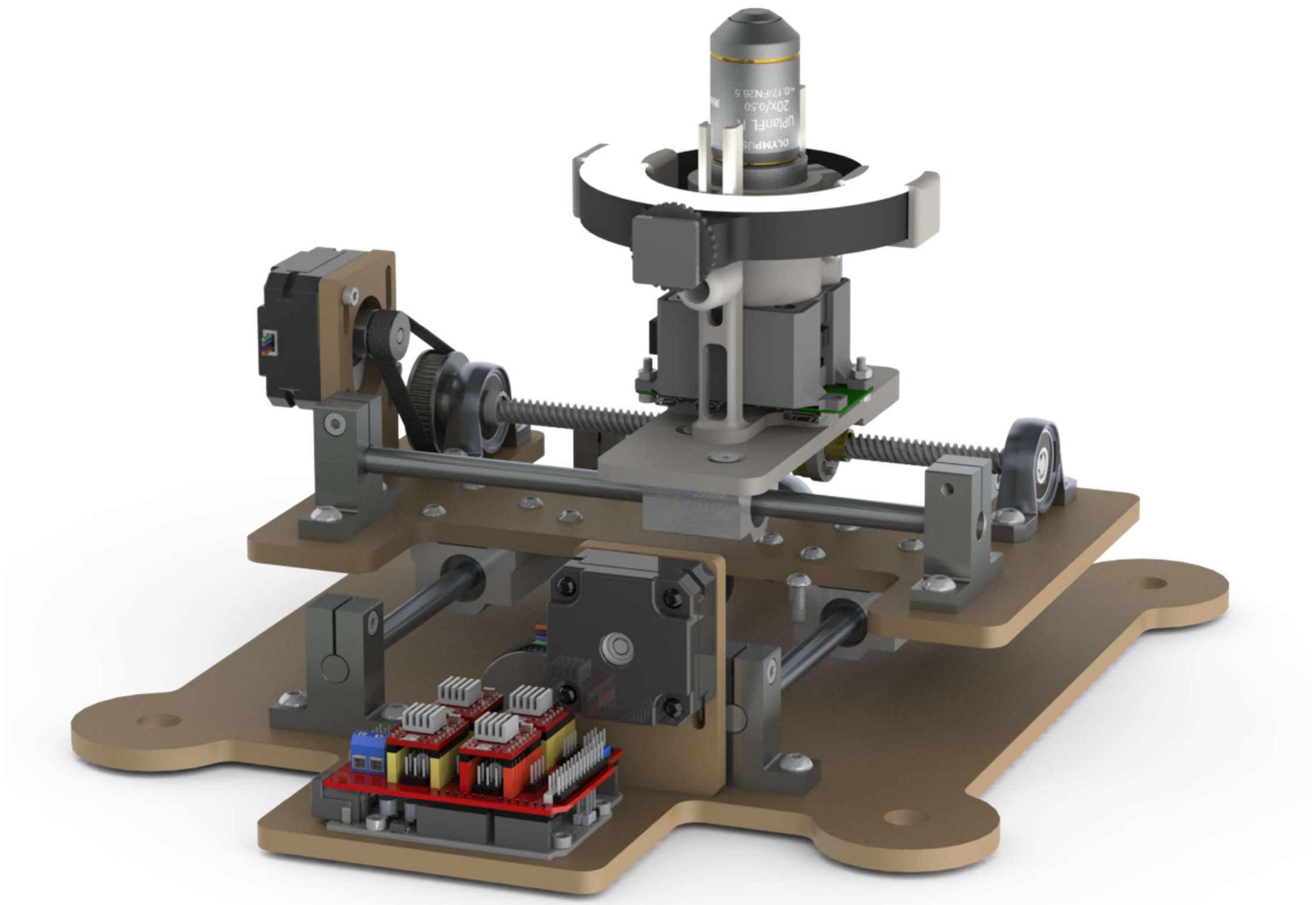

A. Optics Module

Parts

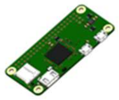
- 1x Raspberry Pi Zero

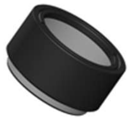
- 1x SM1L05 Lens Tube

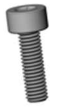
- 2x M3-10 Socket Head Bolt

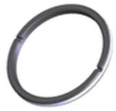
- 1x SM1RR Ring

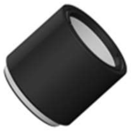
- 1x SM1L10 Lens Tube

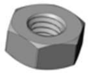
- 2x M3 Nut

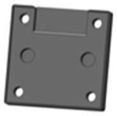
- 1x 3D Printed Top Case

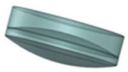
- 1x Achromatic Doublet Lens

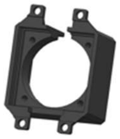
- 1x 3D Printed Bottom Case

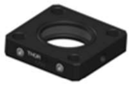
- 1x Cage Plate

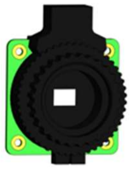
- 1x Raspberry Pi HQ Camera

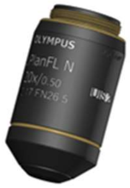

- 1x Objective Lens

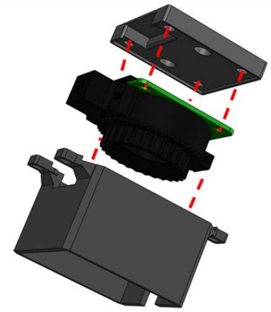	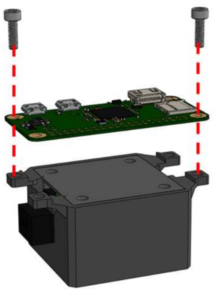	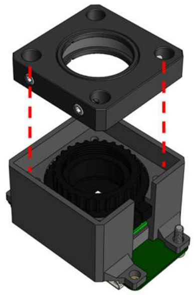
1. Press the Raspberry Pi HQ Camera inside the two 3D printed pieces	2. Attach the Raspberry Pi Zero with two M3 bolts. Attach nuts after building	3. Press the cage plate into the sensor side of the camera
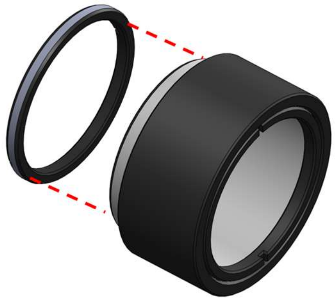	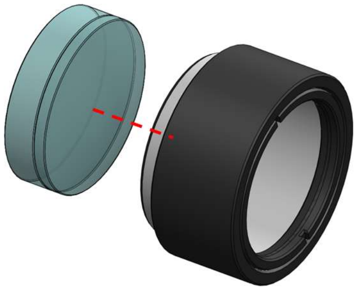	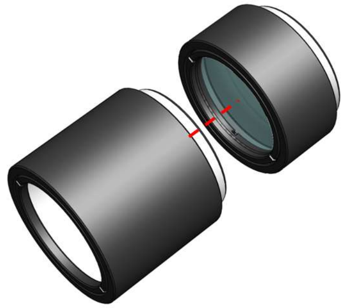
4. Screw the retaining ring midway into 0.5” lens tube.	5. Place the lens into lens tube, convex face facing down.	6. Screw the 1” lens tube to the 0.5” lens tube. Be sure to screw onto the side where the convex face of the lens is.

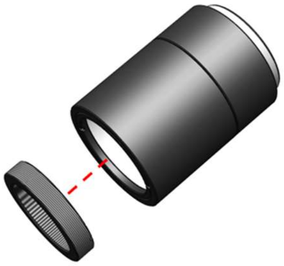	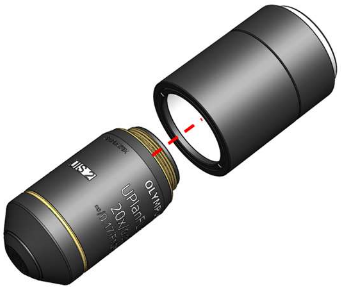	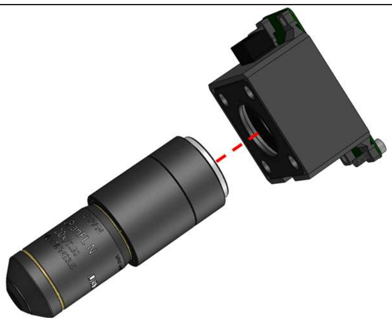
7. Screw a retaining ring into the lens tube set	8. Screw an objective lens to the lens tube set	9. Screw the lens system to the cage plate

B. Z-Axis Module

Parts

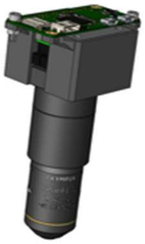

- 1x Optics Module

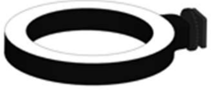

- 1x Ring Light

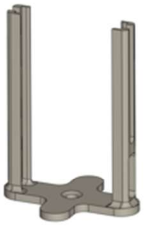

- 1x 3D Printed Stand

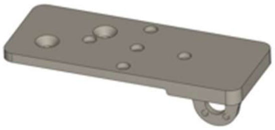

- 1x 3D Printed Base

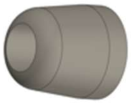

- 2x 3D Printed Knobs

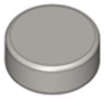

- 10x 6mm Neodymium Magnets

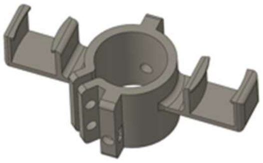

- 1x 3D Printed Combined Hold

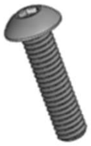

- 4x M4-14 Flat Head Bolt

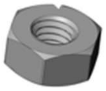

- 4x M4 Nut

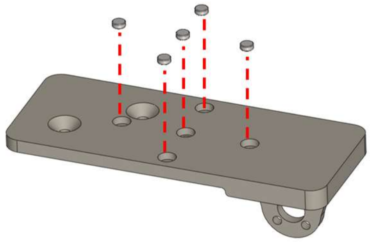	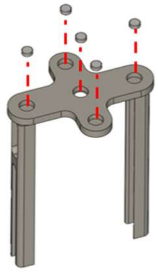	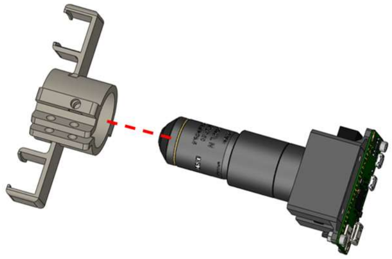
1. Glue 5 magnets into the base portion (ensure magnet polarity is the same)	2. Glue 5 magnets into the stand portion (ensure magnet polarity is the same and opposite of base magnets)	3. Slide the optics unit into the combined hold
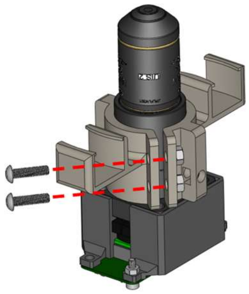	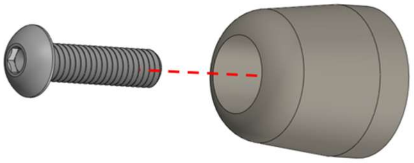	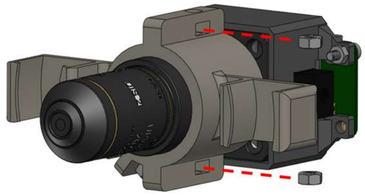
4. Clamp the optics with 2 M4 bolts and nuts	5. Glue an M4 bolt into a knob. (repeat twice for the second knob)	6. Slide 2 M4 nuts into the gaps in the combined hold piece
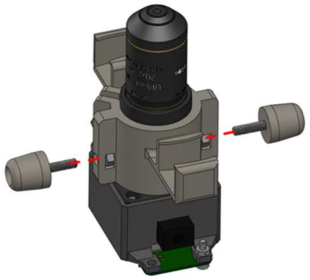	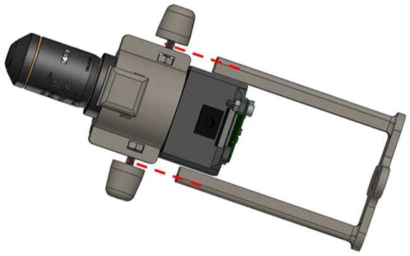	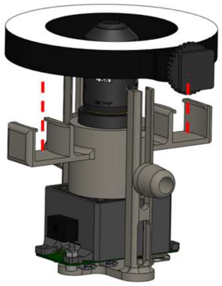
7. Screw the knobs in slightly till they grasp onto the nut	8. Slide the combined module into the stand and screw in the knobs till the system is secure	9. Snap the ring light into the module

C. Y-Axis Module

Parts

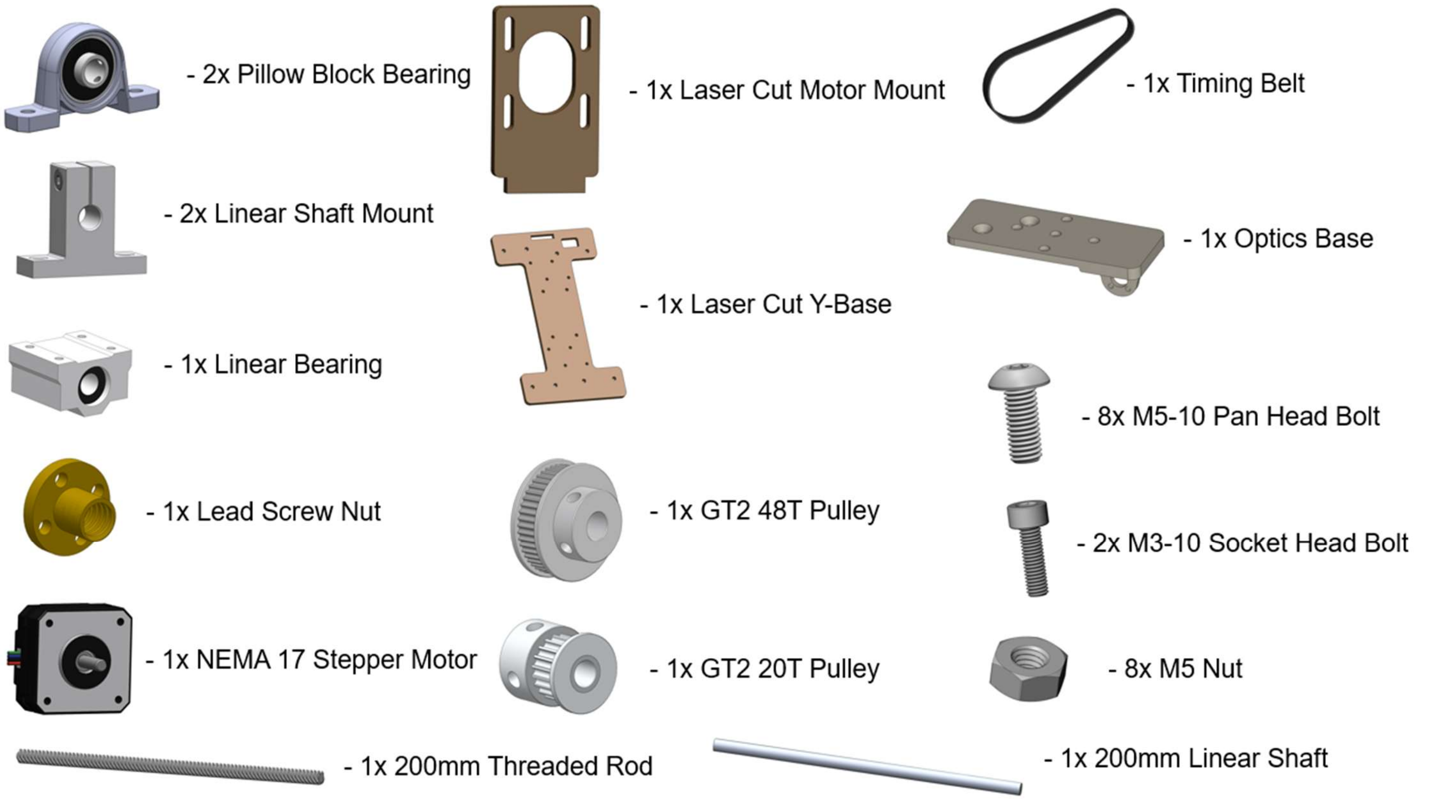

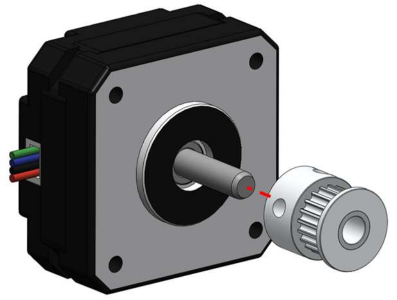	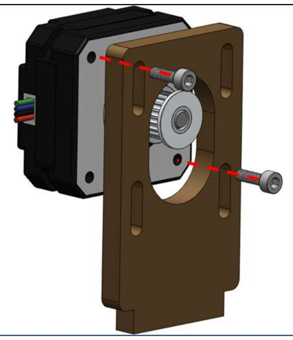	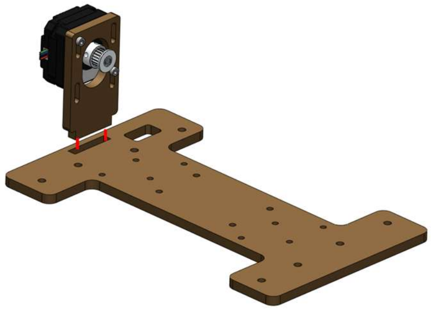
1. Slide the 20T pulley onto stepper motor shaft. Tighten the two set screws on the pulley.	2. Screw the stepper motor onto the laser cut motor mount.	3. Glue the motor mount into the laser cut y-axis base. Use super glue or epoxy. Let fully cure.
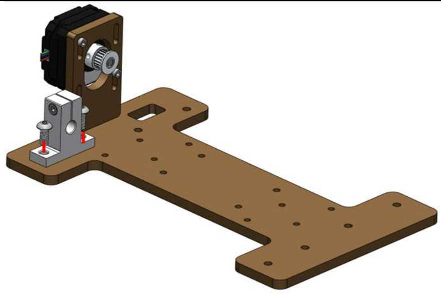	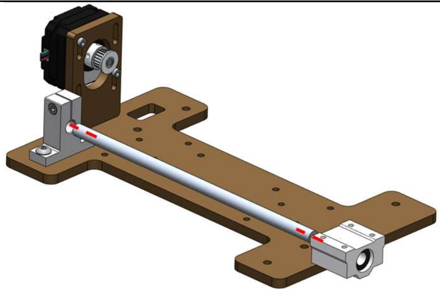	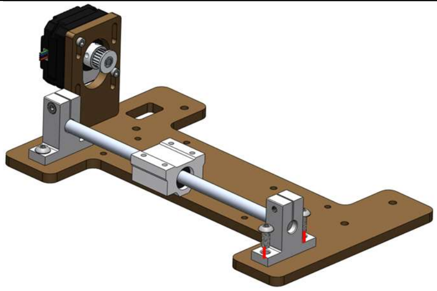
4. Screw a linear shaft mount onto the base with M5 bolts.	5. Slide the linear shaft into the mount and slide a bearing onto the shaft.	6. Screw a linear shaft mount onto the base with M5 bolts.

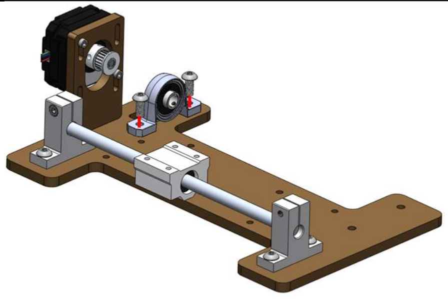	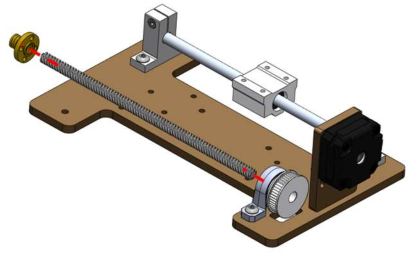	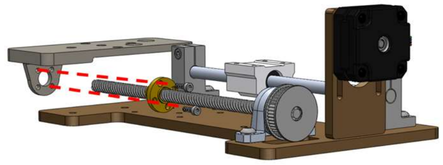
7. Screw a pillow block bearing onto the base with M5 bolts.	8. Place the belt on the 42T pulley and slide the threaded rod through the pillow block bearing and the pulley. Spin the lead nut onto the rod.	9. Screw the optics base to the lead screw nut using two M3 nuts and bolts.
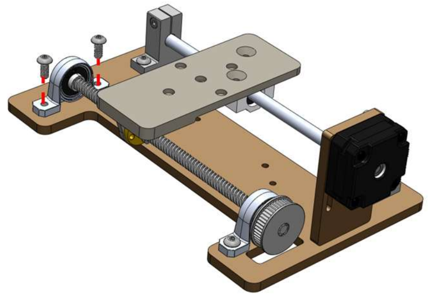	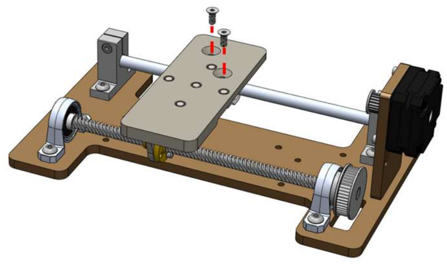	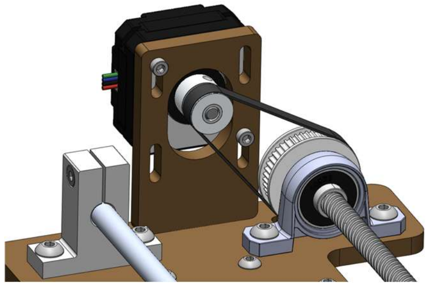
10. Attach the pillow block bearing onto the base with M5 nuts and bolts.	11. Attach the optics base to the linear bearing with two M4 nuts.	12. Adjust the motor mount screws to maintain slight tension in the belt.

D. X-Axis Module

Parts

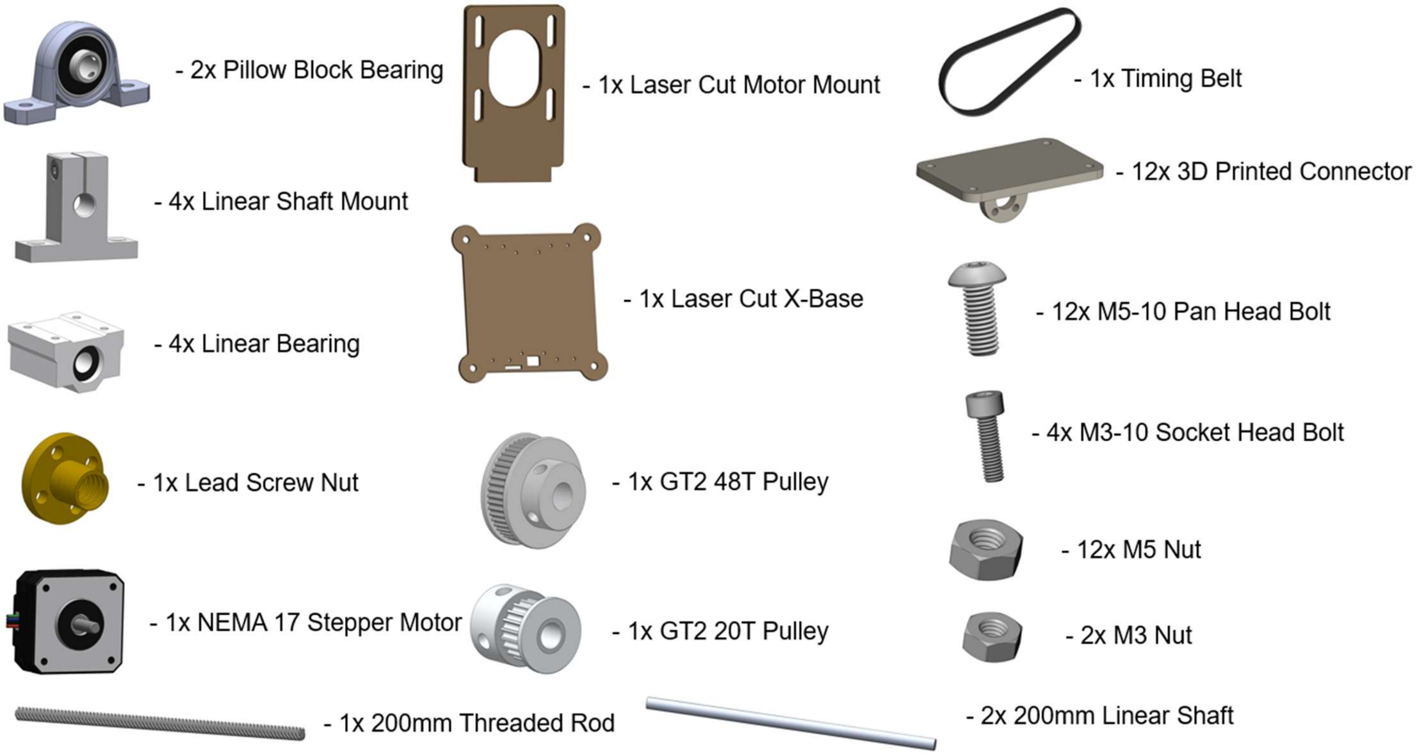

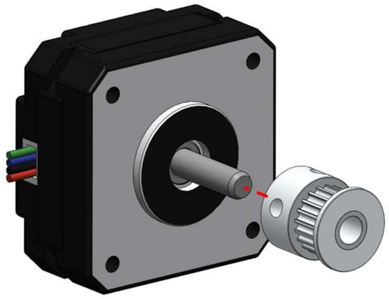	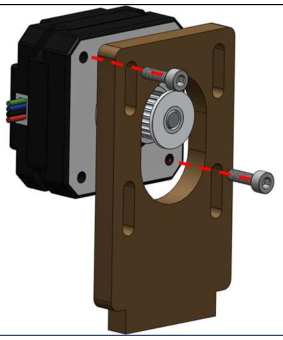	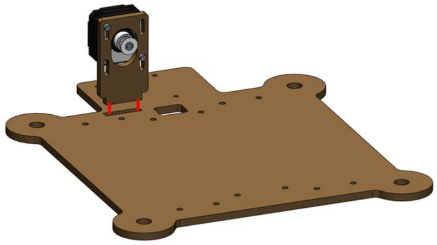
1. Slide the 20T pulley onto stepper motor shaft. Tighten the two set screws on the pulley.	2. Screw the stepper motor onto the laser cut motor mount.	3. Glue the motor mount into the laser cut x-axis base. Use super glue or epoxy. Let fully cure.
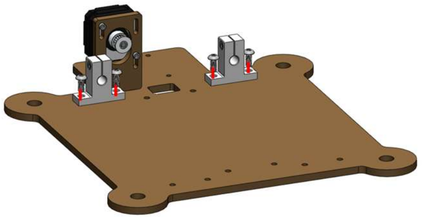	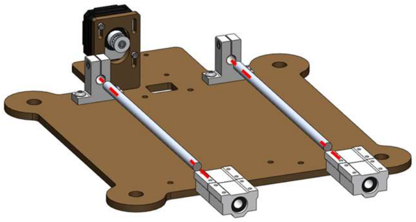	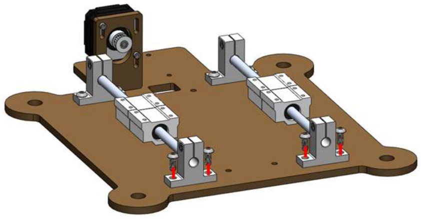
4. Screw two linear shaft mount onto the base with M5 bolts.	5. Slide the linear shafts into the mounts and slide two bearings onto each shaft.	6. Screw two linear shaft mount onto the base with M5 bolts. Tighten the set screws in all linear shaft mounts.

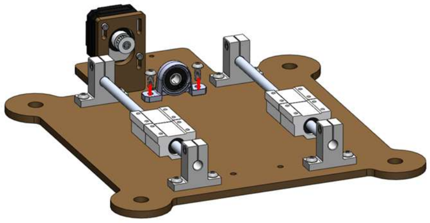	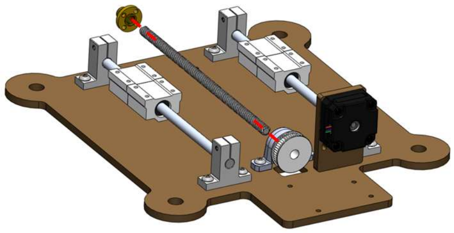	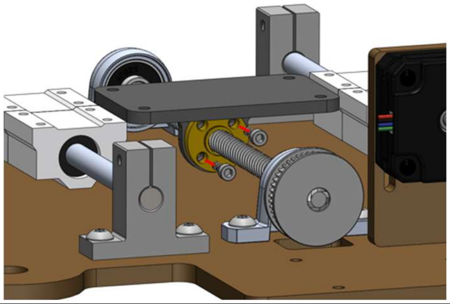
7. Screw a pillow block bearing onto the base with M5 bolts.	8. Place the belt on the 42T pulley and slide the threaded rod through the pillow block bearing and the pulley. Spin the lead screw nut onto the rod.	9. Slide the XY connector onto the threaded rod and secure onto the lead screw nut with 2 M3 bolts and 2 M3 nuts.
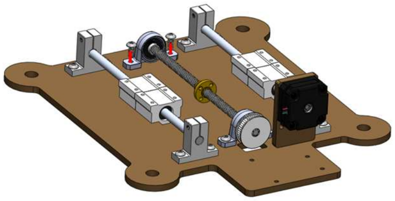	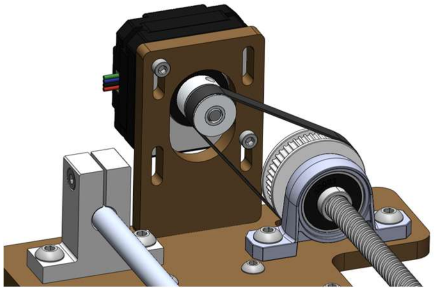	
10. Screw a pillow block bearing onto the base with M5 bolts. Tighten the set screws on the pillow block bearings.	11. Adjust the motor mount screws to maintain slight tension in the belt.	

E. Overall Assembly

Parts

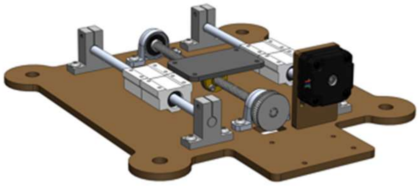

- 1x X-Axis Module

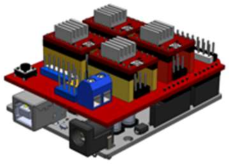

- 1x Arduino Uno + CNC Shield

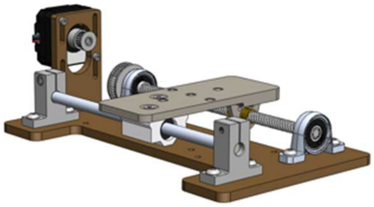

- 1x Y-Axis Module

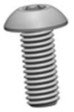

- 14x M4-12 Socket Head Bolt

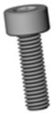

- 4x M2-14 Socket Head Bolt

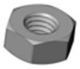

- 4x M3 Nut

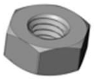

- 4x M4 Nut

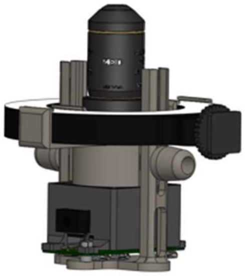

- 1x Z-Axis Stand

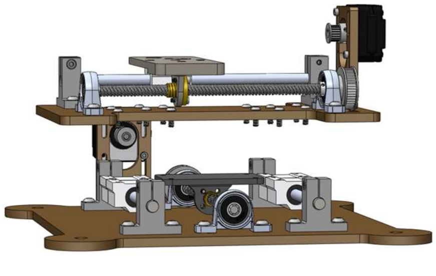	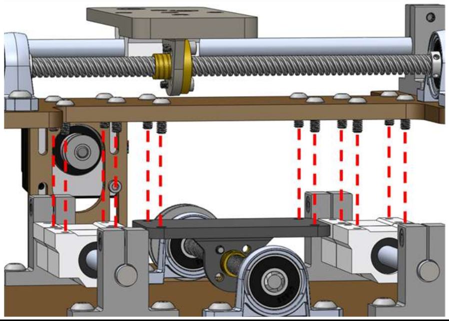	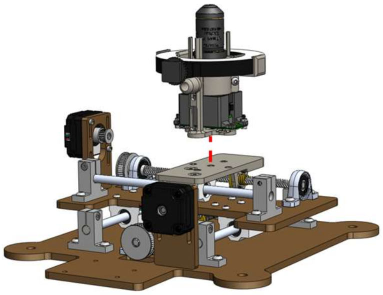
1. Place 12 M4 bolts into the holes on the y-base. If needed place the included spacers between the x and y axes.	2. Screw the bolts into the x-axis linear bearings. Back the bolts in the xy-connector with M4 bolts.	3. Snap the optics z-axis stand onto the y-axis.
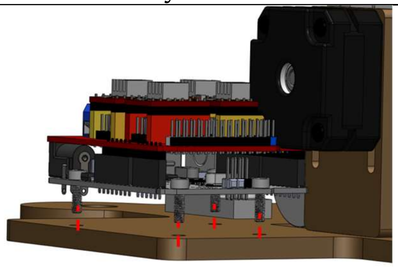	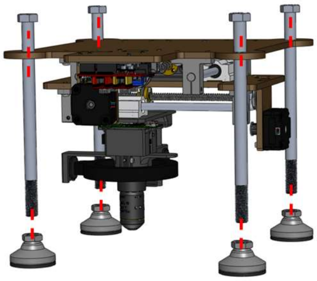	
4. Screw the Arduino Uno to the x-base with 4 M3 bolts.	5. Add support legs to the actuator if needed.	
