# Supplementary material for: Trackoscope: A low-cost, open, autonomous tracking microscope for long-term observations of microscale organisms
Source: PLoS One. 2024 Jul 11;19(7):e0306700. doi: 10.1371/journal.pone.0306700 (PMC11239018; doi:10.1371/journal.pone.0306700)
Supplement: Attachment — Submitted filename: Trackoscope Rebuttal Document.pdf [file pone.0306700.s013.pdf]

We would like to thank the editor and reviewers for the time and effort invested into reviewing our manuscript. All the comments and suggestions provided in the review document have been an invaluable resource for improving our manuscript. In our revision, we have done our best to resolve all concerns brought up by the reviewers.

Please find a point-by-point response to the reviewer's comments below. All changes made to the original manuscript can be found highlighted in the provided marked-up document. In the main manuscript, we have added additional data for multicellular organisms and included dark field illuminated video. We have also added clarification on lighting and tracking accuracy.

Minor changes have been made throughout the manuscript to address grammatical errors. Supplementary videos of organism tracks have been re-organized to accommodate for new multicellular organism data and Figure 2 has been modified to add a dark field image.

In the rebuttal below, the reviewer's comments are in black, the author's comments are in blue, and revised sections of the manuscript addressing the comments are in italicized blue.

Responses to comments from the Editor:

1. When submitting your revision, we need you to address these additional requirements.

Please ensure that your manuscript meets PLOS ONE's style requirements, including those for file naming. The PLOS ONE style templates can be found at

https://journals.plos.org/plosone/s/file?id=wjVg/PLOSOne_formatting_sample_main_body.pdf and

https://journals.plos.org/plosone/s/file?id=ba62/PLOSOne_formatting_sample_title_authors_affiliations.pdf.

Thank you for this feedback, we have corrected notation to match these.

2. Please note that PLOS ONE has specific guidelines on code sharing for submissions in which author-generated code underpins the findings in the manuscript. In these cases, all author-generated code must be made available without restrictions upon publication of the work. Please review our guidelines at https://journals.plos.org/plosone/s/materials-and-software-sharing#loc-sharing-code and ensure that your code is shared in a way that follows best practice and facilitates reproducibility and reuse.

Our code is accessible on GitHub publicly and is updated there. Data is available on figshare.

3. Thank you for stating the following financial disclosure:

[NIH Grant R35GM142588; NIGMS SEPA Grant R25GM142044; NSF Grants MCB-1817334; CAREER IOS-1941933; and the Open Philanthropy Project].

Please state what role the funders took in the study. If the funders had no role, please state: ""The funders had no role in study design, data collection and analysis, decision to publish, or preparation of the manuscript.""

If this statement is not correct you must amend it as needed.

Please include this amended Role of Funder statement in your cover letter; we will change the online submission form on your behalf.

This statement is correct, and we have added it to our cover letter.

4. Thank you for stating the following in the Acknowledgments Section of your manuscript:

[M.S.B. acknowledges funding support from NIH Grant R35GM142588; NIGMS SEPA

Grant R25GM142044; NSF Grants MCB-1817334; CAREER IOS-1941933; and the

Open Philanthropy Project. We thank all members of the Bhamla Lab for their

feedback; Johnathan O'Neil for help setting up Deeplabcut

We note that you have provided funding information that is not currently declared in your Funding Statement. However, funding information should not appear in the Acknowledgments section or other areas of your manuscript. We will only publish funding information present in the Funding Statement section of the online submission form.

Please remove any funding-related text from the manuscript and let us know how you would like to update your Funding Statement. Currently, your Funding Statement reads as follows:

[NIH Grant R35GM142588; NIGMS SEPA Grant R25GM142044; NSF Grants MCB-1817334; CAREER IOS-1941933; and the Open Philanthropy Project]

Please include your amended statements within your cover letter; we will change the online submission form on your behalf.

The statement has been removed from the acknowledgements section and amended within the cover letter.

5. We note that Figure 1, 2ABCD, 3ABC, 4ABCD, 5ABE, S2, S4 and Trackoscope Assembly Instructions A,B,C, D AND E in your submission contain copyrighted images. All PLOS content is published under the Creative Commons Attribution License (CC BY 4.0), which means that the manuscript, images, and Supporting Information files will be freely available online, and any third party is permitted to access, download, copy, distribute, and use these materials in any way, even commercially, with proper attribution. For more information, see our copyright guidelines: http://journals.plos.org/plosone/s/licenses-and-copyright.

We require you to either (1) present written permission from the copyright holder to publish these figures specifically under the CC BY 4.0 license, or (2) remove the figures from your submission:

All figures were created by us and do not contain any copyrighted images.

1. You may seek permission from the original copyright holder of Figure 1, 2ABCD, 3ABC, 4ABCD, 5ABE, S2, S4 and Trackoscope Assembly Instructions A,B,C, D AND E to publish the content specifically under the CC BY 4.0 license.

We recommend that you contact the original copyright holder with the Content Permission Form (http://journals.plos.org/plosone/s/file?id=7c09/content-permission-form.pdf) and the following text:

“I request permission for the open-access journal PLOS ONE to publish XXX under the Creative Commons Attribution License (CCAL) CC BY 4.0 (http://creativecommons.org/licenses/by/4.0/). Please be aware that this license allows unrestricted use and distribution, even commercially, by third parties. Please reply and provide explicit written permission to publish XXX under a CC BY license and complete the attached form.”

Please upload the completed Content Permission Form or other proof of granted permissions as an ""Other"" file with your submission.

In the figure caption of the copyrighted figure, please include the following text: “Reprinted from [ref] under a CC BY license, with permission from [name of publisher], original copyright [original copyright year].”

This is not applicable as all figures were created by us and contain original data.

2. If you are unable to obtain permission from the original copyright holder to publish these figures under the CC BY 4.0 license or if the copyright holder’s requirements are incompatible with the CC BY 4.0 license, please either i) remove the figure or ii) supply a replacement figure that complies with the CC BY 4.0 license. Please check copyright information on all replacement figures and update the figure caption with source information. If applicable, please specify in the figure caption text when a figure is similar but not identical to the original image and is therefore for illustrative purposes only.

Not applicable as figures are created by us.

6. Please remove your figures from within your manuscript file, leaving only the individual TIFF/EPS image files, uploaded separately. These will be automatically included in the reviewers’ PDF.

All figures have been removed from manuscript files and the individual image files have been uploaded.

7. Thank you for uploading your study's underlying data set. Unfortunately, the repository you have noted in your Data Availability statement does not qualify as an acceptable data repository according to PLOS's standards.

At this time, please upload the minimal data set necessary to replicate your study's findings to a stable, public repository (such as figshare or Dryad) and provide us with the relevant URLs, DOIs, or accession numbers that may be used to access these data. For a list of recommended repositories and additional information on PLOS standards for data deposition, please see https://journals.plos.org/plosone/s/recommended-repositories.

We have used figshare to upload and link to our code database. GitHub is still used for the code as it implements version control and the figshare links to it.

figshare.com/projects/Trackoscope_A_Low-Cost_Open_Autonomous_Tracking_Microscope_for_Long-Term_Observations_of_Microscale_Organisms/200934 and github.com/bhamla-lab/Trackoscope

8. Please review your reference list to ensure that it is complete and correct. If you have cited papers that have been retracted, please include the rationale for doing so in the manuscript text, or remove these references and replace them with relevant current references. Any changes to the reference list should be mentioned in the rebuttal letter that accompanies your revised manuscript. If you need to cite

a retracted article, indicate the article's retracted status in the References list and also include a citation and full reference for the retraction notice.

We have reviewed references. Three references (15,28,29) have been added regarding the addition of brine shrimp tracks and an additional frugal device paper.

Responses to comments from Reviewer 1:

Dear Authors, the designed system is well described in the manuscript, and all the documents uploaded to GitHub are accessible in a usable format. Firstly, the range of the Trackoscope which is designed motorized X and Y stages with an 18 cm x 18 cm travel range, is excellent. Additionally, the micro-stepping mechanism is a very useful setup for the device. The Optical Core is designed with adequate quality, and not requiring a high-performance GPU when tracing organisms with OpenCV's Channel and Spatial Reliability Tracker (CSRT tracker) makes the design very efficient.

We would like to thank the reviewer for their feedback and appreciation for the tracking range and simpleness.

In terms of the Performance Evaluation of Tracking and Imaging Capabilities, the resolution for slow-motion organisms is good. However, the resolution for fast organisms is comparatively lower than that of other systems. Nevertheless, it's important to note that there is currently no setup available, except yours, that can effectively trace these organisms at both large spatial and time scales.

Thank you for the feedback, we acknowledge this and note that this resolution is due to the versatility and simpleness of the CSRT and the low-cost actuators.

Please correct the species name in the supplementary files to italic and small-capital letters.

Thank you for pointing this out, we have adjusted the species names accordingly.

For Trackoscope's Customizable Design for Wider Use: I recommend adding an additional supplementary file to the manuscript, demonstrating the tracing of a multicellular organism like Botryllid ascidian (marine invertebrate) larvae or adult oral siphon movements (such as feeding behavior) or zooplankton movements. This addition could attract wider attention from the scientific community.

We thank the reviewer for making this suggestion. We have already included tardigrades as a multicellular organism. But we agree that adding other multicellular organisms will attract wider attention. Therefore, we have also conducted tracks with brine shrimp, a type of zooplankton, and have added a supplementary video showcasing a track of brine shrimp nauplii in darkfield.

Tracking of brine shrimp nauplii emphasizes their chaotic and random movements. In the nauplii stage, brine shrimp are still adjusting to movement with front jointed antennae and their motion is not smooth as their phyllopods (swimming appendages) have not developed yet [27]. In the track, we observe circular lurching motion [28] as the nauplii moves at an average speed of 515 µm/s with a top speed of 1200 µm/s (1.2 body lengths/second) (S6 Video). As the nauplii attempts to move linearly, it tends to move in a clockwise circle suggesting that its left antenna is stronger than the right; when coordination is achieved, fast linear motion is observed. (lines 216-223, page 8)

Responses to comments from Reviewer 2:

This is excellent paper demonstrating the design of a cheap and simply to build tracking microscope. It seems to me that a lot of thought has been given to its design and execution.

I highly recommend the publication of this manuscript.

We thank the reviewers for their kind words and appreciation for the device.

I have one comment: The final tracks look a bit rough. The tracking dot moves around on the organism a lot. This can be fixed in post-processing I suppose? I.e. the video is saved (using Open Broadcast Studio) and then subsequent analysis could fine-tune the tracking precision. It would be nice if such a process could be demonstrated, but is definite not necessary for the publication of this manuscript. (more of a nice to have, than need to have).

We acknowledge that the tracks are slightly rough. This is due to the simple and versatile nature of the OpenCV CSRT as it constantly adapts its model throughout the track from the user's initial 1 frame selection. This adaptation over the course of the track can lead to the tracking dot shifting around on the organism as observed. However, with the versatility and adaptability of the tracker, the tracking dot tends to stay on the organism, even though it is not always the center of the organism. We have added this clarification to the paper. You are correct in assuming that this can be corrected in post-processing, but this would require a separate custom software that takes in the video and position output from Trackoscope to then adjust the tracking values based on the video.

The CSRT model updates the bounding box by continually adapting over the duration of the track as it considers both the appearance of the organism and its motion characteristics. This adaptability makes the CSRT particularly effective in handling challenges such as occlusion, rotation, and scale variation. (lines 96-99, page 5)

Additionally, as the CSRT model evolves over the track, the perceived center of the organism may not always be accurate or precise; this is another factor for the incorporation of a central zone as the perceived center, while always on the organism, may not be on the exact center. (lines 106-109, page 5)

Responses to comments from Reviewer 3:

Priya Soneji and authors describe Trackoscope, an automated tracking microscope enabling long-duration observations of motile microorganisms. The authors highlight limitations of conventional microscopes in studying behaviors of swimming microorganisms over extended time periods. They introduce a novel tracking microscope design utilizing low-cost, modular hardware components and custom (open) software implementing open-source computer vision algorithms. The system demonstrates impressive microscopic resolution down to 8.77 µm and adaptable speed tracking from 0.02 to 11.8 body lengths/sec across diverse organisms with unique behaviors. They limit their exploration to 2D, and discuss the complexity and cost associated with motorized Z stages-while showcasing the data accessible in these dimensions. Examples showcase insights into feeding, morphology changes, reproduction, and comparative locomotion. As an open, flexible platform made with economical parts, I believe Trackoscope promises to make automated microscopy more accessible for research and education.

We would like to thank the reviewer for the time and effort required to provide such detailed comments, especially on the low-cost open nature of Trackoscope, its resolution, and overall design.

Comments:

The Trackoscope design fits well into the growing ecosystem of open hardware/software instrumentation. Trackoscope provides functionality comparable to expensive commercial systems at a fraction of the cost, while utilizing open libraries such as OpenCV and others in the Python universe that enable customization without reliance on proprietary software. The integration of hardware and software components to quickly enable versatile automated tracking of cells will be useful to many researchers and educators.

The resolution analysis confirms capabilities for resolving microorganism cells and structures. Speed profiling indicates suitable performance across crawling, swimming, and ciliates with rapidly changing behavior.

Diverse organism tracking experiments highlight new behavioral insights uniquely enabled by Trackoscope's combination of resolution, field of view, and duration.

An accessible and customizable platform promotes adoption by researchers with limited budgets. Open design fosters educational uses and customization. The discussion of multiple low-cost materials such as MDF makes the project adaptable to many communities.

We thank the reviewer for their appreciation of the modularity of Trackoscope, and its tracking versatility.

Minor Comments:

Additional details on tracking accuracy, effect of illumination, and computational analysis methods would further strengthen characterizations of organism behavior. As the system seems catered to ciliates, the integration of a simple dark field illumination could be uniquely beneficial for these studies with Trackoscope. This could significantly improve visualization of low-contrast transparency features in protists, like pellicles, membranes, and cytoskeletal elements. This is a minor comment however, as the system is designed with frugality in mind, and the simplicity of the illumination setup is understandable- the open and customizable optics design of Trackoscope allows flexibility in illumination methods, future iterations could investigate more imaging modes.

We appreciate the reviewer's feedback. We agree that additional illumination modes would allow for better visibility. We have added data with dark field illumination for Spirostomum and Brine Shrimp in supplementary videos 2, 3, and 5 and have modified Figure 2 to include an example of darkfield illumination to visibly show the effect of illumination.

Dark field illumination can also be used with Trackoscope by adding a 3D printed attachment to the ring light that directs the light around the sample from below. (lines 80-82, pages 4-5)

Dark field imaging allows for higher contrast when observing more translucent organisms (Fig 2c and S2 Video). (lines 128-129, page 6)

Regarding the comment about tracking accuracy, we found that such a metric is hard to quantify. The OpenCV CSRT constantly adapts its model over the duration of the track leading to some error. So while

the perceived center will still be on the organism’s body, it may not always be the precise center. We have added this clarification to the paper as well as more detail on the adaptability of the tracker.

Additionally, as the CSRT model evolves over the track, the perceived center of the organism may not always be accurate or precise; this is another factor for the incorporation of a central zone as the perceived center, while always on the organism, may not be on the exact center. (lines 106-109, page 5)

Figures are clear and simple, I could not find anything that requires major improvement-besides the request that the text in the plot legends be increased in size (as much as possible) for clarity in reading. The plot ticks in the amoeba data in figure 5 are especially small.

We thank the reviewer for this feedback and have increased the font size in Figure 5.

Overall Evaluation:

I recommend this manuscript for publication. The authors have designed, validated, and demonstrated a novel tracking microscope that overcomes limitations of conventional designs. Unique insights into microorganism locomotion and behaviors are enabled. The open, flexible architecture promises to expand access to automated microscopy. The integration of the many powerful python-based computer vision libraries, while discussing the benefits of simplified methods such as CSRT, are appreciated. While machine learning techniques like convolutional neural networks have revolutionized computer vision, they also have disadvantages like requiring large training datasets, extensive computational resources, and loss of interpretability. Trackoscope displays how efficient deployment of robust open-source tools can sidestep these issues and provide reliable tracking performance without complexity. Avoiding sophisticated neural networks in favor of classical CV algorithms aligns well with the overall aims of an accessible, adaptable platform. This work represents a valuable contribution with both scientific and educational impacts in the microscopy field.

We thank the reviewer for their encouraging comments and their time.
